# Differential Activation of Splenic cDC1 and cDC2 Cell Subsets following Poxvirus Infection of BALB/c and C57BL/6 Mice

**DOI:** 10.3390/cells13010013

**Published:** 2023-12-20

**Authors:** Lidia Szulc-Dąbrowska, Zuzanna Biernacka, Michał Koper, Justyna Struzik, Małgorzata Gieryńska, Ada Schollenberger, Iwona Lasocka, Felix N. Toka

**Affiliations:** 1 Department of Preclinical Sciences, Institute of Veterinary Medicine, Warsaw University of Life Sciences-SGGW, 02-786 Warsaw, Poland; zuzanna_biernacka@sggw.edu.pl (Z.B.); justyna_struzik@sggw.edu.pl (J.S.); malgorzata_gierynska@sggw.edu.pl (M.G.); ada_schollenberger@sggw.edu.pl (A.S.); 2 Center for Integrative Mammalian Research, Ross University School of Veterinary Medicine, Basseterre P.O. Box 334, Saint Kitts and Nevis; 3 Institute of Genetics and Biotechnology, Faculty of Biology, University of Warsaw, 02-106 Warsaw, Poland; m.koper@uw.edu.pl; 4 Department of Biology of Animal Environment, Institute of Animal Science, Warsaw University of Life Sciences-SGGW, 02-786 Warsaw, Poland; iwona_lasocka@sggw.edu.pl

**Keywords:** dendritic cell, cDC1, cDC2, ectromelia virus, Th immune response

## Abstract

Conventional dendritic cells (cDCs) are innate immune cells that play a pivotal role in inducing antiviral adaptive immune responses due to their extraordinary ability to prime and polarize naïve T cells into different effector T helper (Th) subsets. The two major subpopulations of cDCs, cDC1 (CD8α^+^ in mice and CD141^+^ in human) and cDC2 (CD11b^+^ in mice and CD1c^+^ in human), can preferentially polarize T cells toward a Th1 and Th2 phenotype, respectively. During infection with ectromelia virus (ECTV), an orthopoxvirus from the *Poxviridae* family, the timing and activation of an appropriate Th immune response contributes to the resistance (Th1) or susceptibility (Th2) of inbred mouse strains to the lethal form of mousepox. Due to the high plasticity and diverse properties of cDC subpopulations in regulating the quality of a specific immune response, in the present study we compared the ability of splenic cDC1 and cDC2 originating from different ECTV-infected mouse strains to mature, activate, and polarize the Th immune response during mousepox. Our results demonstrated that during early stages of mousepox, both cDC subsets from resistant C57BL/6 and susceptible BALB/c mice were activated upon in vivo ECTV infection. These cells exhibited elevated levels of surface MHC class I and II, and co-stimulatory molecules and showed enhanced potential to produce cytokines. However, both cDC subsets from BALB/c mice displayed a higher maturation status than that of their counterparts from C57BL/6 mice. Despite their higher activation status, cDC1 and cDC2 from susceptible mice produced low amounts of Th1-polarizing cytokines, including IL-12 and IFN-γ, and the ability of these cells to stimulate the proliferation and Th1 polarization of allogeneic CD4^+^ T cells was severely compromised. In contrast, both cDC subsets from resistant mice produced significant amounts of Th1-polarizing cytokines and demonstrated greater capability in differentiating allogeneic T cells into Th1 cells compared to cDCs from BALB/c mice. Collectively, our results indicate that in the early stages of mousepox, splenic cDC subpopulations from the resistant mouse strain can better elicit a Th1 cell-mediated response than the susceptible strain can, probably contributing to the induction of the protective immune responses necessary for the control of virus dissemination and for survival from ECTV challenge.

## 1. Introduction

Poxviruses entered an infamous page in the history of mankind due to the variola virus (VARV), which caused one of the most devastating and widespread diseases (smallpox), characterized by high mortality, high transmissibility, and high infectivity, leaving its victims with ugly scars and/or blindness [1]. Although smallpox can be considered a historic disease due to its eradication through a successful vaccination campaign, zoonotic poxviruses are now a growing public health problem [2]. As an example, the recent 2022 global outbreak of human monkeypox, caused by monkeypox virus (MPXV), may indicate changes in the biologic aspects of the virus, leading to its expansion from endemic regions of Central and Western Africa [3].

The outcome of a viral disease is controlled by many host immune cells, including dendritic cells (DCs), due to their ability to activate antiviral immune mechanisms and direct T cells toward distinct T helper (Th) immune responses [4]. DCs constitute a heterogeneous population of cells in terms of functionality, phenotype, and location, which is the basis for their different classifications [5,6,7]. In the murine spleen, the two major populations of resident conventional DCs (cDCs) are distinguished based on the expression of cell surface molecules, i.e., CD8α^+^CD11b^−^ (cDC1) and CD8α^−^CD11b^+^ (cDC2), which are found mostly in the marginal zone and the T cell areas of periarterial lymphoid sheath (PALS), respectively [7]. In addition to typical markers presented on all cDCs, such as CD11c and major histocompatibility class II (MHC II), cDC1 also express subtype-specific and cross-species-conserved markers, such as X-C motif chemokine receptor 1 (XCR1) and C-type lectin receptor DNGR-1 (dendritic cell NK lectin group receptor 1) (also known as CLEC9A) (Table 1). Moreover, cDC1 express CD205 and CD207, but these two molecules are also found on other cells [8]. In contrast, cDC2 broadly express signal regulatory protein α (SIRPα) and CD11b, but the cDC2 subtype found in lymphoid tissues (e.g., spleen) can also express the CD4 molecule [8,9,10]. The cDC1 and cDC2 subpopulations are also functionally distinct. cDC1 are potent activators of CD8^+^ T cells through cross-presentation and stimulate T cell differentiation toward Th1 cells [11,12,13], whereas cDC2 are mainly specialized in the activation of CD4^+^ T cells and Th2 differentiation [14,15].

One of the best small animal models to study pathogenesis of smallpox and other generalized poxvirus infections is ectromelia virus (ECTV)—the causative agent of mousepox, a disease that is the mouse homolog of human smallpox [16]. Similarities between mousepox and smallpox include the low viral dose required for infection and the general infection progression, which initially bypasses lung involvement but later presents a characteristic exanthematous rash. Differences lie in the disease course kinetics, which are shorter in mousepox, and the extent of necrosis in primary target organs; mousepox shows extensive necrosis in the liver and spleen, unlike smallpox, which does not exhibit extensive necrosis in the liver, bone marrow, and lymph nodes. It has been shown that during early stages of mousepox (2–5 days post-infection, dpi) susceptible and resistant strains of mice generate different types of Th immune response in lymphoid organs, such as dLNs and spleens. Susceptible BALB/c and A/J mice preferentially generate a Th2 immune response, associated with an absent, weak, or delayed cytotoxic T lymphocyte (CTL) response resulting in the development of full-blown mousepox and death, whereas the resistant C57BL/6 mice develop a Th1 immune response accompanied by strong CTL activity and recovery from a primary ECTV infection [17]. Despite the predispositions of C57BL/6 and BALB/c mice to exhibit Th1- and Th2-dominant immune responses, respectively, in other infectious [18,19,20], as well as non-infectious [21,22] diseases, the engagement of cDCs subpopulations in stimulation of a different Th immune response in resistant and susceptible mouse strains during ECTV infection remains unresolved. The capacity of cDC subsets to direct Th1/Th2 polarization during infectious diseases is adaptable, varying with factors like stimulation conditions and antigen dose, and is linked to genetically determined resistance or susceptibility to the infection. Therefore, mouse interstrain differences in cDCs’ ability to “switch” the type of the Th immune response during infection can ultimately determine the course of the disease (Table 2).

In the context of poxvirus infection, different DC subsets may participate in establishing a protective immune response necessary for survival of the infected host from the primary infection [27,28,29,30], as well as in the pathogenesis of poxvirus infections [31]. However, the role of different subsets of cDCs in the polarization of T cells during mousepox has not been elucidated. It remains unknown how cDC1 and cDC2 subsets stimulate the Th1 or Th2 responses in C57BL/6 and BALB/c mice during mousepox. More importantly, the comparative study of the in vivo influence of ECTV on immune functions of cDC subsets in BALB/c and C57BL/6 mice, which may have an important role in determining the direction of host immune response, has not been assessed. Therefore, in the present study, we set to understand the potential role of cDC subsets in influencing Th responses in mice susceptible and resistant to ECTV infection. Our results show that ECTV infects both subsets of splenic cDCs in resistant C57BL/6 and susceptible BALB/c mice, but cDC subsets from BALB/c mice exhibit a higher viral load than that form C57BL/6 mice during the early stages of mousepox (5 dpi). Interestingly, at 5 dpi, cDCs from both mouse strains are able to mature and become activated; however, cDC1 and cDC2 from BALB/c mice exhibit a higher maturation and activation status than that of their counterparts among C57BL/6 mice. Despite this higher activation status, both subsets of cDCs from BALB/c mice produce lower amounts of Th1-polarizing cytokines, such as IL-12 and IFN-γ, compared to those produced by their C57BL/6 counterparts. Lastly, although the cDC1 and cDC2 are able to mature, infection with ECTV leads to a decline in the ability of the cDC subsets of both mouse strains to stimulate proliferation and the production of Th1- and Th2-polarizing cytokines by allogeneic CD4^+^ T cells of C3H mice, compared to that of DC subsets of uninfected animals. Such an inhibitory effect is more pronounced in cDCs originating from susceptible BALB/c mice. Overall, our data indicate that both cDC subsets in C57BL/6 mice are more potent in stimulating a Th1 immune response than their BALB/c counterparts are. Therefore, resistant strains of mice are likely to develop a more robust cell-mediated immune response and recover from lethal mousepox.

## 2. Materials and Methods

### 2.1. Virus

In all experiments, the Moscow strain of ECTV purchased from American Type Culture Collection (Manassas, VA, USA) (ATCC, VR-1374) was used. The propagation, purification, and titration of ECTV were performed, as previously described [32].

### 2.2. Animals

Male inbred mice, aged 8–12 weeks, from the BALB/c (H-2d), C57BL/6 (H-2b), and C3H (H-2k) strains, were acquired from the animal facility at the Maria Sklodowska-Curie Memorial Cancer Centre and Institute of Oncology in Warsaw, Poland. These mice were housed in a climate-controlled facility where the temperature was maintained at 24 ± 2 °C and the relative humidity was maintained at 50 ± 10%. They had continuous access to regular chow and water. All experimental procedures involving these mice received approval from the 3rd Ethical Committee for Animal Experimentation at the Warsaw University of Life Sciences—SGGW (permission no. 34/2012), adhering to the institutional Guidelines for Care and Use of Laboratory Animals. Additionally, the animal facilities at the Faculty of Veterinary Medicine, which hold the registration number 14313537, have full certification from the district veterinary inspector.

BALB/c and C57BL/6 mice received injections in their hind footpads with 200 plaque-forming units (PFU) of ECTV, administered in a solution of phosphate-buffered saline (PBS). Each injection was 10 μL per footpad. As a negative control, mice were injected with sterile PBS. After 2, 4, 7 and/or 14 days post-infection (dpi) mice were sacrificed under anesthesia and the spleen was used for cell preparation.

### 2.3. Preparation of Single-Cell Suspension of Splenocytes

Single-cell suspensions from spleens of BALB/c and C57BL/6 mice were prepared via enzymatic digestion, as previously described [33]. For immunophenotyping, splenocyte suspensions were obtained using collagenase D digestion, whereas for the isolation of DC subsets, cell suspensions were prepared using liberase blendzyme II digestion followed by magnetic selection. Briefly, spleens were injected with Hanks’ balanced saline solution (HBSS) with Ca^2+^ and Mg^2+^ (Sigma-Aldrich, St. Louis, MO, USA) containing 100 U/mL of collagenase D or 100 Mandl U/mL of liberase blendzyme II and 20 µg/mL of DNAse I grade II (all from Roche Applied Science, Mannheim, Germany). Spleens were then mechanically disrupted with a needle and the obtained tissue fragments were vigorously pipetted and treated with 400 U/mL of collagenase or 400 Mandl U/mL of liberase solutions. After 60 min of incubation at 37 °C, tissue fragments were pipetted and strained through a nylon mesh to release single cells. Next, the cell suspensions were passed through 30 µm pre-separation filters (Miltenyi Biotec, Auburn, CA, USA), and after erythrocyte depletion with Red Blood Cell Lysis Buffer (Sigma-Aldrich), the splenocytes were counted using a Neubauer hemocytometer. Cell viability was assessed using a trypan blue exclusion assay.

### 2.4. Multi-Color Immunophenotyping

The phenotype and maturation of DC subsets were assessed using flow cytometry with up to ten colors of fluorescence. Freshly prepared single-cell suspensions of splenocytes were incubated for FcR blocking with anti-mouse CD16/CD32 monoclonal antibody (mAb) clone 2.4G2 (BD Biosciences, San Jose, CA, USA) for 15 min on ice and then placed into BD Horizon Brilliant Stain Buffer (BD Biosciences) containing an antibody cocktail. The following mAbs were used in appropriate combinations: anti-CD11c-BV421 clone N418 (BioLegend, San Diego, CA, USA), anti-CD11b-BV605 clone M1/70, anti-CD4-APC-H7 clone GK1.5, anti-CD8a-PerCP-Cy5.5 clone 53-6.7, anti-CD45R/B220-BV786 clone RA3-6B2, (all from BD Biosciences), anti-CD205(DEC-205)-PE/Cy7 clone NLDC-145, anti-XCR1-BV785 clone ZET, anti- CD172a (SIRPα)-PE clone P84 (all from BioLegend), anti-H-2D[b]-PE clone KH95, anti-I-A/I-E-BV711 clone M5/114.15.2, anti-H-2D[d]-PE clone 34-2-12, anti-CD83-PE clone Michel-19, anti-CD40-APC clone 3/23, anti-CD80-APC clone 16-10A1, anti-CD86-BV711 clone GL-1 (BD Biosciences), and anti-I-A/I-E-PerCP clone M5/114.15.2 (BioLegend). After a 30 min incubation at room temperature (RT), the cells were washed with phosphate-buffered saline (PBS) and then analyzed using flow cytometry. To rule out non-specific antibody binding, the cells were stained with corresponding isotype controls obtained from BD Biosciences or BioLegend. Furthermore, for defining gating boundaries, Fluorescence Minus One (FMO) samples were used as negative controls for each marker.

### 2.5. Intracellular Cytokine Assay

Splenocytes were re-stimulated with ionomycin (1 µg/mL) and phorbol 12-myristate 13-acetate (PMA; 500 ng/mL), both sourced from Sigma-Aldrich, for 6 h. During the last 5 h of this culture period, Brefeldin A (6 µg/mL from BD Biosciences) was added. The cells were then incubated with CD16/CD32 monoclonal antibody (mAb) for 15 min at 4 °C, followed by staining with monoclonal antibodies (mAbs) for surface markers for 30 min at 4 °C. After this, the cells were fixed and permeabilized using Cytofix/CytoPerm (BD Biosciences) for 25 min at 4 °C. Finally, the cells were stained intracellularly with appropriate mAbs or corresponding isotype controls (BD Biosciences) for 30 min at 4 °C. The antibodies used for intracellular staining experiments included the following: IL-12(p40/p70)-APC (clone C15.6), IFN-γ-PE (clone XMG1.2), MCP-1-PE (clone 2H5), IL-10-APC (clone JES5-16E3), TNF-FITC (clone MP6-XT22), IL-2-PE (clone JES6-5H4) IL-4-APC (clone 11B11), anti-IL-17A-PE (clone TC11-18H10) (all from BD Biosciences), and CCL3-PE (clone DNT3CC) (eBioscience, San Diego, CA, USA). After washing, the cells were analyzed using a flow cytometer.

### 2.6. Flow Cytometry Analysis

**Figure 1:**
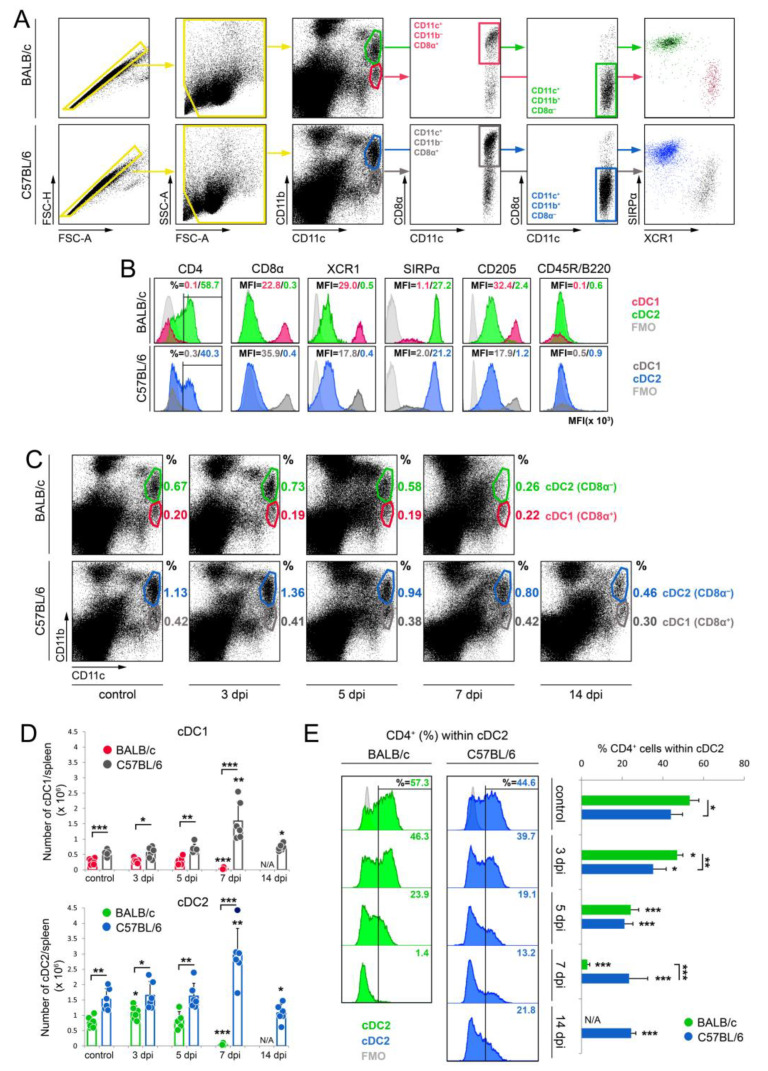
ECTV infection decreasing the number of cDC1 and cDC2 subsets in the spleen of susceptible BALB/c mice. (**A**) Gating strategy for the evaluation of cDC1 (CD11b^−^CD11c^+^ indicated by red and grey circles) and cDC2 (CD11b^+^CD11c^+^ indicated by green and blue circles) subsets in spleens of BALB/c and C57BL/6 mice, respectively, based on the expression of CD11b and CD11c markers. (**B**) The percentage of indicated marker-positive cells or the surface expression (shown as mean fluorescent intensity (MFI)) of the indicated markers by cDC1 (CD11b^−^CD11c^+^) and cDC2 (CD11b^+^CD11c^+^) subsets. Light grey histograms represent fluorescence minus one (FMO) controls. (**C**) The percentage of cDC1 (CD11b^−^CD11c^+^) and cDC2 (CD11b^+^CD11c^+^) subsets in spleens of BALB/c and C57BL/6 mice during ECTV infection. Circles depict gates and the numbers are the percentage of cells within a particular gate. (**D**) The total number of cDC1 and cDC2 per spleen of BALB/c and C57BL/6 during ECTV infection. The dots represent the number of a particular cell population in an individual animal counted per total number of live splenocytes. The mean values for the groups (*n* = 5–6) are shown in columns and the standard deviations are indicated by the error bars. Significant differences were estimated and are compared to the control group unless otherwise indicated by horizontal bars between two columns (* *p* < 0.05, ** *p* < 0.01, and *** *p* < 0.001). (**E**) The percentage of CD4^+^ cells within the cDC2 subpopulation in spleens of BALB/c and C57BL/6 mice infected with mousepox. Light grey histograms indicate FMO controls. In all cases, the percentage/MFI values shown are of the representative cytogram/histogram, *n* = 6 mice/group, with one animal comprising one flow cytometry sample. The mean values for the groups (*n* = 5–6) are shown in columns and the standard deviations are indicated by the error bars. Significant differences were estimated and are compared to the control group unless otherwise indicated by horizontal bars between two columns (unpaired Student’s *t*-test: * *p* < 0.05, ** *p* < 0.01, and *** *p* < 0.001). N/A—not analyzed.

The cytometry analysis was performed using a BD LSR Fortessa apparatus with BD FACSDiva 7.0 software (Becton Dickinson and Company, Heidelberg, Germany). To guarantee the consistent and unprejudiced analysis of samples within different experiments, the application setting module was applied after instrument characterization and performance tracking using the Cytometer Setup and Tracking (CST) system. Acquired events were plotted against forward scatter (FSC) height (FSC-H) and FSC area (FSC-A) parameters to discriminate single cells from doublets. Side scatter (SSC) A and FSC-A characteristics were used to exclude debris and create a gate for live splenocytes (Figure 1A). Up to 1 × 10^6^ events were collected for each experimental sample.

### 2.7. Isolation of Splenic cDC1 (CD8α^+^) and cDC2 (CD8α^−^) Subsets

In some experiments, freshly obtained splenocytes were subjected to the magnetic cell separation system (MACS) for the positive selection of the cDC1 (CD8α^+^) population using CD8^+^ Dendritic Cell Isolation Kit (Miltenyi Biotec), in accordance with the manufacturer’s instructions. During the first step of separation, the splenocytes were depleted of T, B, and NK cells using a cocktail of biotinylated antibodies against CD90.2, CD45R, and CD49b, followed by incubation with anti-biotin MicroBeads. Next, the remaining cells were incubated with anti-CD8α beads for 30 min on ice and then passed through MS columns. The collected unlabeled cells were further proceeded for cDC2 (CD8α^−^) subset isolation using CD11c beads. The purity of the DC subsets was assessed via flow cytometry and remained higher than 93%.

### 2.8. Fluorescence Microscopy

Splenic CD8α^+^ and CD8α^−^ DC subsets we analyzed for the presence of CD8α, CD11b, and/or ECTV antigens. The cells were stained with appropriate primary antibodies conjugated either with PE or FITC for 60 min. DNA was stained with 1 μg/mL of Hoechst 33342 (Calbiochem, San Diego, CA, USA) for 2.5 min. Slides were mounted in ProLong Gold Antifade Reagent (Invitrogen Life Technologies, Carlsbad, CA, USA). After staining, the cells were examined using an Olympus BX60 fluorescence microscope equipped with CAM-UC90 Camera Set (Olympus, Tokyo, Japan). Image analysis was performed using CellSens Dimension 1.9 software (Olympus, Tokyo, Japan).

### 2.9. Plaque Forming Assay

A plaque-forming assay, previously detailed in reference [32], was conducted as follows. Vero cell monolayers, cultured in 24-well plates, were exposed to 10-fold serial dilutions of ECTV stocks. These stocks were derived from CD8α^+^ and CD8α^−^ dendritic cell (DC) subsets, isolated from the spleens of C57BL/6 and BALB/c mice. After a period of 5 days, the Vero cell monolayers were stained with 0.3% crystal violet, allowed to air-dry, and then the plaques were counted using an Olympus IX71 inverted microscope (Olympus, Tokyo, Japan).

### 2.10. RNA Isolation

High Pure RNA Isolation Kit (Roche Diagnostics GmbH, Mannheim, Germany) was used to isolate total RNA, in accordance with the manufacturer’s instructions. The concentration and purity of isolated RNA from cDC1 and cDC2 subsets were assessed by measuring the optical density (OD) ratio at 260 and 280 nm using a Take-3 system on an Epoch BioTek spectrophotometer and analyzed in Gen5 1.11 software (BioTek Instruments, Inc., Winooski, VT, USA). The A260/A280 ratio was above 2. The quality of the RNA was assessed in terms of RNA degradation using RNA Nano Chips on Agilent Bioanalyzer 2100 (Agilent Technologies, Palo Alto, CA, USA). The RNA integrity number (RIN) values were equal to or higher than 8.7.

### 2.11. RT-qPCR

Transcriptor First Strand cDNA Synthesis Kit from Roche Diagnostics GmbH was used to reverse-transcribe 550 ng of RNA into cDNA, following the manufacturer’s instructions. For analysis, selected genes involved in the regulation of dendritic cell (DC) maturation, activation, and function were examined using the RealTime ready Custom Panel, also from Roche Diagnostics GmbH. This panel includes assays for 92 target genes and 4 reference genes: Rn18s, Gusb, Gapdh, and Hsp90ab1. Rn18s and Gusb were identified as the most stable reference genes across all experimental groups and were therefore selected as appropriate housekeeping genes. Each RT-PCR assay included primers and a pre-plated Universal ProbeLibrary (UPL) Probe in the wells of the plate. Furthermore, five controls were incorporated into each qPCR run: three positive controls targeting different sections of the same transcript (the 3′ end, the middle, and the 5′ end), and two negative controls to check for the presence of genomic DNA. All controls were conducted in triplicate. The reaction mixture, comprising 550 ng of cDNA and the LightCycler 480 Probes Master mix from Roche Diagnostics GmbH, was prepared and distributed into 384-well plates. Real-time PCR was then carried out using the LightCycler 480 instrument, also from Roche Diagnostics GmbH.

Amplification was performed at 95 °C for 10 s, 60 °C for 30 s, and 72 °C for 1 *s* during 45 cycles, following an initial pre-incubation at 95 °C for 10 min. The 2^−ΔΔCt^ method was applied to calculate the fold change of each target gene expression, relative to the geometric mean mRNA expression of 2 housekeeping genes: Gapdh and Hsp90ab1.

### 2.12. Ingenuity Pathway Analysis (IPA)

IPA analysis (Qiagen, Germantown, MD, USA) was applied to determine interaction networks between genes using core analysis. Transcripts with differences greater than 2-fold (*p* ≤ 0.05) found between cDC1 and cDC2 from control and ECTV-infected BALB/c and C57BL/6 mice were input into IPA.

### 2.13. Mixed Leukocyte Reaction (MLR)

BALB/c and C57BL/6 splenic CD8α^+^ and CD8α^−^ DC subsets were co-cultured with the C3H CD4^+^ T cells obtained, as previously described [34], for 5 days in a ratio of 1:5 (4 × 10^4^ DC: 2 × 10^5^ T cells) in a 96-well plate in triplicate. To evaluate T cell proliferation, CD4+ T cells were pre-labeled with 0.5 mM CFSE (carboxyfluorescein diacetate succinimidyl ester, from Sigma-Aldrich) for 10 min at 37 °C before cell co-culture. As a positive control for T cell activation, CD4+ T cells incubated with 1 µg/mL of concanavalin A (Con A, also obtained from Sigma-Aldrich) were used.

For the single-cell level evaluation of cytokine production, CD4^+^ T cells were re-stimulated with Iono + PMA in the presence of Brefeldin A, and stained intracellularly for IFN-γ, TNF, IL-2, IL-4, IL-10, and IL-17A (as described in Section 2.5).

### 2.14. Cytokine ELISA

The levels of IFN-γ, TNF, IL-2, IL-4, IL-10, and IL-17A in the culture supernatants from dendritic cell (DC)/T-cell co-cultures were measured using BD OptEIA ELISA kits from BD Biosciences, following the manufacturer’s instructions. Optical densities were measure using Epoch Microplate Spectrophotometer (BioTek Instruments, Inc., Winooski, VT, USA) at 450 nm. The cytokine concentrations were determined by interpolating them from a linear calibration curve, which was conducted alongside the sample measurements in the same assay.

### 2.15. Statistical Analysis

Data are presented as the mean ± standard deviation (SD) based on at least three independent experiments, typically involving 2 mice per experiment, resulting in a total of 5 to 6 mice per experimental group. The distribution of variables was assessed using the Shapiro–Wilk W-test. Depending on the distribution, either the two-independent (unpaired) Student’s *t*-test (for normally distributed data) or the Mann–Whitney U-test (for non-normally distributed data) was applied. Additionally, some data were analyzed using either a two-dependent (paired) Student’s *t*-test (for normal distribution) or the Wilcoxon signed-rank test (for non-normal distribution). Statistical analyses were conducted using STATISTICA 13.1 software (StatSoft Inc., Tulsa, OK, USA), with statistical significance determined at levels of * *p* < 0.05, ** *p* < 0.01, and *** *p* < 0.001.

## 3. Results

### 3.1. The Percentage and the Number of cDC1 and cDC2 Subsets in Spleens of Susceptible BALB/c Mice Decreases during the Acute Phase of Mousepox

cDC1 and cDC2 subsets of cDCs were distinguished based on the surface expression of CD11c, CD11b, CD205, CD45R/B220, CD4, and CD8α molecules, as previously reported [35,36], with some modifications. Firstly, singlet cells were selected and distinguished from doublet cells on the basis of FSC-H vs. FSC-A plots, and debris and dead cells were removed via SSC-A vs. FSC-A gating (Figure 1A). The cells were then evaluated according to CD11b^+^ and CD11c^+^ expression, and two populations of CD11c^+^ cells were identified: CD11c^+^CD11b^−^ and CD11c^+^CD11b^+^. In the CD11c^+^CD11b^−^ and CD11c^+^CD11b^+^ compartments, CD8α^+^ and CD8α^−^ cells were selected, respectively. As shown on Figure 1B, CD11c^+^CD11b^−^CD8α^+^ cells had no expression of CD4 and CD45R/B220, and were CD205^high^, XCR1^+^, and SIRPa–; therefore, such cells correspond to cDC1 cells. Meanwhile, CD11c^+^CD11b^+^CD8α^−^ cells were CD45R/B220^−^, CD205^low^, XCR1–, and SIRPa^+^, and on average, 53% and 44% of these cells exhibited the expression of a CD4 molecule in BALB/c and C57BL/6 mice, respectively. Therefore, CD11c^+^CD11b^+^CD8α^−^ cells correspond to cDC2 cells.

In general, the percentage and the number of CD11c^+^CD11b^−^CD8α^+^ cells (referred to as cDC1) and CD11c^+^CD11b^+^CD8α^−^ cells, including CD4^+^ cells (referred to as cDC2) were higher in spleens of uninfected C57BL/6 mice compared to that in uninfected BALB/c mice (Figure 1C–E). A similar relationship was maintained throughout the infection period; however, only in spleens of BALB/c mice were the percentage and the number of cDC2 cells significantly (*p* < 0.05) elevated at 3 days post-infection (dpi). At 7 dpi, the number and the percentage of cDC2 decreased significantly (*p* < 0.001) in BALB/c, but not in C57BL/6 mice. Because at this time post-infection, severe necrosis was observed in spleens of BALB/c mice, the total number of cDC1 per spleen was also significantly (*p* < 0.001) reduced, despite the lack of a decrease in the percentage of these cells. On the contrary, in spleens of C57BL/6 mice the number of both cDC subsets was significantly (*p* < 0.01) elevated at 7 dpi. Due to the severe necrosis of parenchymal organs, BALB/c mice succumbed to disease between 9 and 11 dpi, and, therefore, only spleens of C57BL/6 mice were analyzed at 14 dpi. At this time point, the percentage of cDC1 and cDC2 significantly (*p* < 0.001) decreased, whereas the number of these cells significantly (*p* < 0.05) increased in spleens of C57BL/6 mice (Figure 1C,D).

In mousepox, a gradual loss of CD4^+^ DCs within the cDC2 subpopulation was observed in spleens of both strains of mice (Figure 1E). This observation may be due to a reduction in CD4^+^ DCs within the cDC2 population. Alternatively, it could result from an increased number of monocyte-derived cells that fall within the cDC2 gate, thereby reducing the proportion of CD4^+^ cells. However, further investigation is needed to substantiate this hypothesis. During a later stage of infection (14 dpi), the percentage of CD4^+^ cDC2 began to increase again in spleens of C57BL/6 mice (Figure 1E). Taken together, we show the disparity of cDC subsets numbered in the two mouse strains following infection with ECTV with an incremental advantage in C57BL/6 mice.

### 3.2. cDC Subsets from BALB/c Mice Exhibit Higher Viral Load Than Those from C57BL/6 Mice during Early Stages of Mousepox

**Figure 2:**
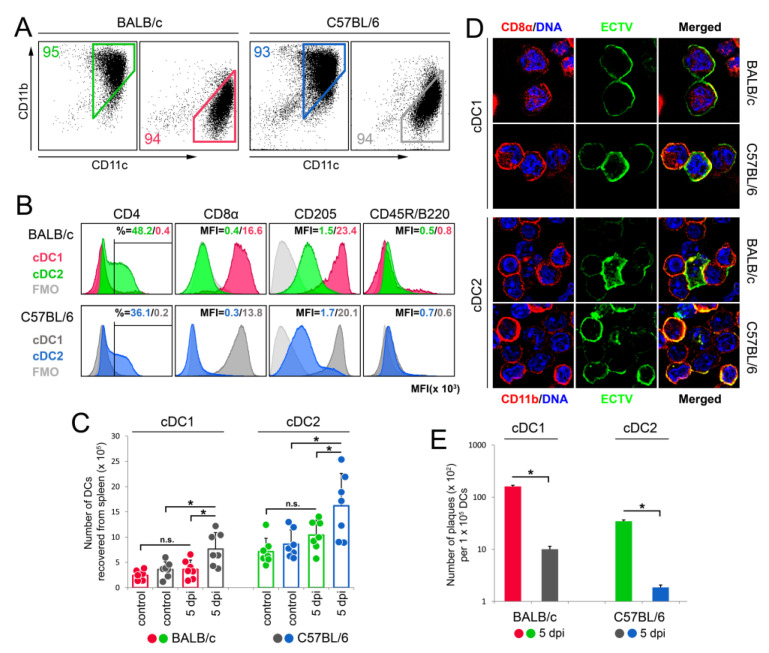
ECTV infects cDC1 and cDC2 in spleen of BALB/c and C57BL/6 mice; however, both cDC subsets of susceptible mice yielded a higher virus titer than that of resistant mice. (**A**) Purity assessment of cDC1 and cDC2 subsets via flow cytometry after magnetic cell separation (MACS). Representative flow cytometry dot plots showing the percentage of cDC1 (CD8α^+^ as CD11b^−^CD11c^+^) and cDC2 (CD8α^−^ as CD11b^+^CD11c^+^) subsets after MACS isolation from spleen of uninfected BALB/c and C57BL/6 mice. Numbers in each gate indicate the percentage of cells positive for given markers. (**B**) The percentage of the indicated marker-positive cells or surface expression (shown as mean fluorescent intensity (MFI)) of the indicated markers by cDC1 (CD11b^−^CD11c^+^) and cDC2 (CD11b^+^CD11c^+^) subsets after MACS enrichment. Light-grey histograms represent fluorescence minus one (FMO) controls. In all cases, percentage/MFI values shown are of the representative cytogram/histogram, *n* = 6 mice/group, with one animal comprising one flow cytometry sample. (**C**) The total number of cDC1 and cDC2 subsets recovered from the spleen of uninfected and infected BALB/c and C57BL/6 mice at 5 dpi after MACS separation. The dots represent the number of a particular cell population in the individual spleen. The mean values for the groups (*n* = 7) are shown in columns and the standard deviations are indicated by the error bars. Significant differences are indicated by horizontal bars between two columns (unpaired Student’s *t*-test: * *p* < 0.05, n.s.—not significant). (**D**) Fluorescence microscopy analysis of the expression of ECTV antigens on MACS-separated cDC1 and cDC2 subsets, stained for the presence of CD8α and CD11b markers, respectively. (**E**) Number of plaques per 1 × 10^5^ splenic cDC1 or cDC2 of BALB/c and C57BL/6 mice at 5 dpi with ECTV. Significant differences are indicated by horizontal bars between two columns (unpaired Student’s *t*-test: * *p* < 0.05).

DC subsets were recovered from spleens of uninfected and ECTV-infected BALB/c and C57BL/6 mice at 5 dpi. Flow cytometry analysis revealed that the purity of recovered cDC1 and cDC2 subsets was more than 93% (Figure 2A). The isolated cDC1 subset from both mouse strains had no CD4 expression but exhibited a high expression of CD8α and CD205 molecules (Figure 2B). In contrast, the cDC2 subset showed the presence of the CD4 molecule, a moderate expression of CD205, and no expression of CD8α. Both cDC subsets did not show the expression of the B220 molecule (Figure 2B).

The number of cDC1 recovered from uninfected BALB/c and C57BL/6 mice was 2.5-fold lower than the number of cDC2 (Figure 2C). At 5 dpi, only in spleens of C57BL/6 mice was there a significant (*p* < 0.05) increase in the number of recovered cDC1 and cDC2, compared to that in uninfected animals. Additionally, at this time point of infection, the number of both cDC subsets in the spleen of C57BL/6 mice was significantly (*p* < 0.05) higher compared to that in ECTV-infected BALB/c mice (Figure 2C).

Next, we analyzed the expression of ECTV antigens on both cDC subsets in infected BALB/c and C57BL/6 mice. Fluorescence microscopy analysis revealed that cDC1 and cDC2 from both mouse strains were stained positive for ECTV antigens on their surface (Figure 2D). To check whether or not these cells can be infected by ECTV, we reisolated the virus from 1 × 10^5^ cDC subsets and found that ECTV can productively replicate within these cells in both mouse strains. However, a higher titer of the virus was detected in cDC1 and cDC2 derived from susceptible BALB/c mice compared to that in resistant C57BL/6 mice (Figure 2E).

### 3.3. cDC Subsets from BALB/c Mice Show Higher Maturation Profile than Those from C57BL/6 Mice during Acute Phase of Mousepox

**Figure 3:**
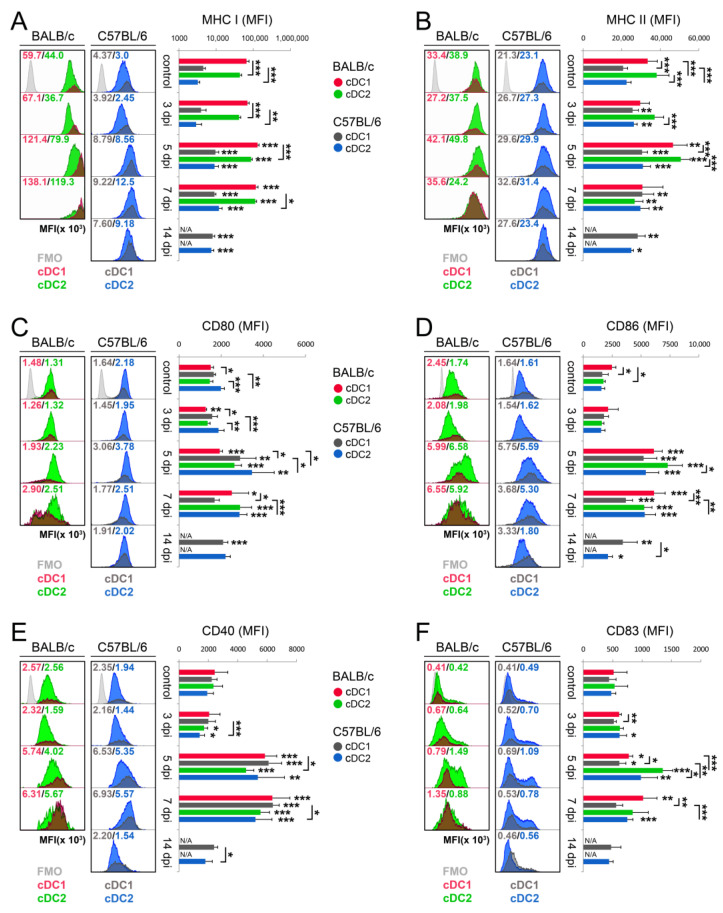
ECTV infection promotes maturation of cDC1 and cDC2 in spleen of BALB/c and C57BL/6 mice during early stages of infection; however, the MHC II expression on cDC subsets drops under severe mousepox in susceptible mice. Surface expression (shown as mean fluorescent intensity (MFI)) of major histocompatibility class I (MHC I) molecules (**A**), MHC II (**B**), CD80 (**C**), CD86 (**D**), CD40 (**E**), and CD83 (**F**) by cDC1 (CD11b^−^CD11c^+^) and cDC2 (CD11b^+^CD11c^+^) subsets. Light-gray histograms represent fluorescence minus one (FMO) controls. In all cases, a representative histogram is shown from *n* = 5 or 6 mice/group, with one animal comprising one flow cytometry sample. The mean values for the groups (*n* = 5–6) are shown in columns and the standard deviations are indicated by the error bars. Significant differences were estimated and compared to the control group unless otherwise indicated by horizontal bars between two columns (unpaired or paired Student’s *t*-test and Mann–Whitney U-test or Wilcoxon signed-rank test: * *p* < 0.05, ** *p* < 0.01, and *** *p* < 0.001). N/A—not analyzed.

There were several intercellular and interstrain differences in the expression of MHC and co-stimulatory molecules on the surface of cDC1 and cDC2 in spleens of uninfected and ECTV-infected BALB/c and C57BL/6 mice. In both uninfected mouse strains, the level of MHC I and MHC II expression was significantly (*p* < 0.001) higher and lower, respectively, on cDC1 compared to cDC2 (Figure 3A,B). The CD80 and CD86 expression was significantly (*p* < 0.05) higher on cDC2 of the C57BL/6 mouse strain and on cDC1 of the BALB/c mouse strain, respectively, compared to that in the other cDC subset (Figure 3C,D). There were no intercellular differences in the surface level of CD40 and CD83 in both mouse strains (Figure 3E,F).

In the case of cDC1, differences between mouse strains were observed in the expression pattern of MHC II, CD80, and CD86 molecules, whereas only differences in the expression of MHC II were seen in the cDC2 subset (Figure 3). Both cDC subsets from BALB/c mice expressed significantly (*p* < 0.001) higher levels of MHC II molecules compared to that in counterparts among C57BL/6 mice, whereas cDC1 from BALB/c or C57BL/6 mice had a significantly (*p* < 0.05) lower or higher level of CD80 or CD86 molecules, respectively, compared to cells from other mouse strains.

Already at 3 dpi, the expression of MHC II increased significantly (*p* < 0.01) on C57BL/6 cDC1 and cDC2 subsets (Figure 3B), whereas the CD80 level decreased significantly (*p* < 0.01) only on BALB/c cDC1 (Figure 3C). During ECTV infection, both cDC subsets from both mouse strains significantly (*p* < 0.01) upregulated the expression of all analyzed MHC and co-stimulatory molecules at 5 dpi. BALB/c cDC1 cells expressed higher levels of MHC II and CD83, whereas cDC2 cells expressed higher levels of MHC II, CD86, and CD83 compared to that in their C57BL/6 counterparts (Figure 3). The expression of high levels of all analyzed maturation markers on both cDCs subsets from both mouse strains lasted until 7 dpi; however, BALB/c cDC1 showed a significantly (*p* < 0.05) higher expression of CD80, CD86, and CD83 molecules than did C57BL/6 cDC1 (Figure 3). Since BALB/c mice succumbed to disease between 8 and 12 dpi, only cells from C57BL/6 mice were analyzed at 14 dpi. At this time point, the level of MHC and co-stimulatory molecules on both C57BL/6 cDC subsets was mostly elevated but began to fall, and some animals approached the control values. These results show that although the maturation of the cDC subsets does occur following infection with ECTV, the naturally susceptible strain produces highly matured cDCs compared to the resistant strain of mice.

### 3.4. cDC Subsets from C57BL/6 Mice Secrete Higher Levels of Th1-Polarizing Cytokines Than Those from BALB/c Mice in Mousepox

**Figure 4:**
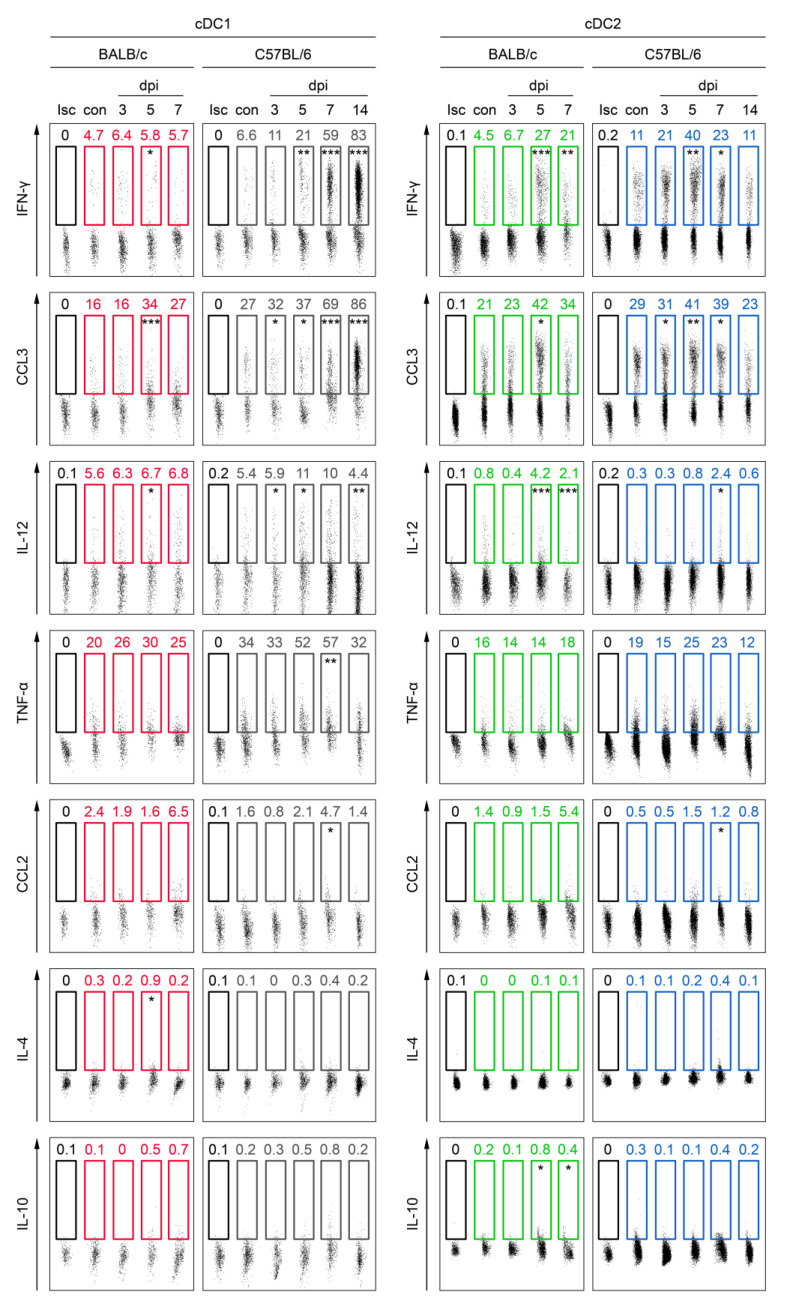
cDC1 and cDC2 of C57BL/6 mice producing higher levels of Th1 cytokines than those of BALB/c mice during ECTV infection. Splenocytes were re-stimulated with Ionomycin + PMA for 6 h in the presence of Brefeldin A for the last 5 h of culture, and then stained intracellularly. Representative flow cytometry dot plots showing the percentage of cDC1 (CD11b^−^CD11c^+^) and cDC2 (CD11b^+^CD11c^+^) subsets producing IFN-γ, CCL3, IL-12, TNF-α, CCL2, IL-4, or IL-10. The numbers within each gate denote the percentage of cells positive for a given marker, derived from one representative dot plot of at least five independent mice. Statistically significant differences between the means of the experimental group (*n* = 5 or 6) and the control group (*n* = 5 or 6) are indicated with asterisks (unpaired Student’s *t*-test or Mann–Whitney U-test: * *p* < 0.05, ** *p* < 0.01, and *** *p* < 0.001). Isc—isotype control.

cDC subsets participate in T cell polarization through the production of different types of Th1 and Th2 cytokines. Therefore, we next investigated the capacity of splenic cDC1 and cDC2 to synthesize IFN-γ, CCL3, IL-12, and TNF-α (Th1-polarizing cytokines) and CCL2, IL-4, and IL-10 (Th2–polarizing cytokines) in mousepox in BALB/c and C57BL/6 mice (Figure 4). In general, both cDC subsets in C57BL/6 mice produced significantly (*p* < 0.05) higher levels of IFN-γ than did their BALB/c counterparts throughout the duration of mousepox infection. The production of IFN-γ by cDC1 and cDC2 from both mouse strains started to increase from 3 dpi. Similarly, levels of CCL3 synthesized by cDC2 subsets from both mouse strains were comparable at 5 dpi. The percentage of IL-12-synthesizing C57BL/6 cDC1 increased significantly (*p* < 0.05) in the early stages of infection (3 and 5 dpi). The percentage of cDC2 producing IL-12 increased significantly (*p* < 0.05) at 7 dpi in spleens of both mouse strains compared to control values. The production of TNF-α by cDC1 and cDC2 from BALB/c mice was slightly elevated at 5 and 7 dpi, respectively, compared to the control values; however, this increase was not statistically significant (*p* > 0.05). cDC1 from C57BL/6 synthesized more TNF-α at 5 and 7 dpi compared to the control, and the difference was statistically significant (*p* < 0.01) at 7 dpi. The production of TNF-α by C57BL/6 cDC2 was slightly but not significantly increased at 5 and 7 dpi compared to control values (Figure 4).

The production of the Th2-polarizing cytokine, CCL2, by cDC1 and cDC2 increased at 7 dpi in both mice strains; however, the increase was statistically significant (*p* < 0.05) only in the case of C57BL/6 mice. Meanwhile, a statistically significant (*p* < 0.05) increase in IL-4 production was only observed in BALB/c cDC1 at 5dpi compared to the control (Figure 4). cDC1 from both mouse strains produced higher levels of IL-10 at 5 and 7 dpi compared to the control, but only in the case of C57BL/6 mice was the increase statistically significant (*p* < 0.05). A similar change was also observed in the case of cDC2 from BALB/c mice, but the increase was not statistically significant (*p* > 0.05) (Figure 4).

### 3.5. cDC Subsets, Especially cDC2, from BALB/c Mice Exhibit More Up-Regulated Genes Engaged in Cell Maturation and Activation Than C57BL/6 cDCs during Early Stages of Mousepox

**Figure 5:**
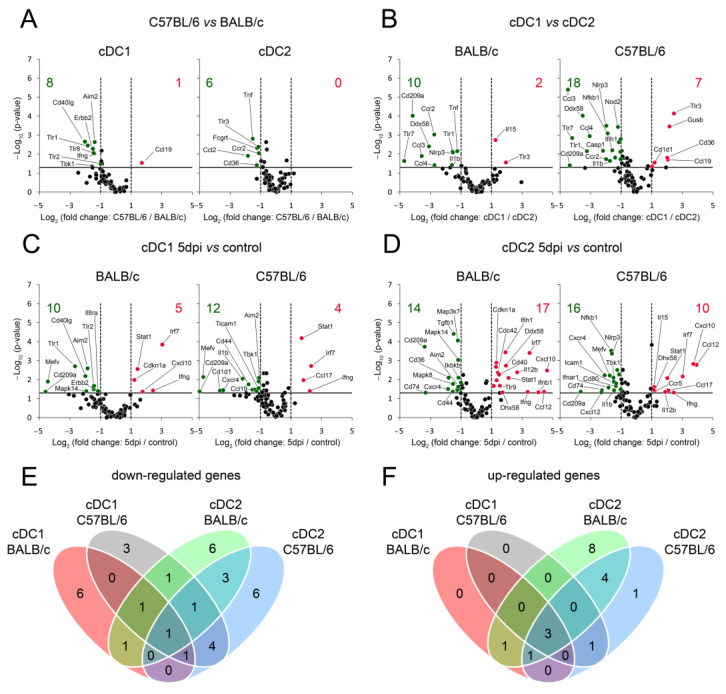
Diagram showing that ECTV induces higher changes in the expression of genes associated with dendritic cell maturation and activation in cDC1 and cDC2 of BALB/c mice than in those of C57BL/6 mice in the early stage of mousepox. The volcano plots depict differentially expressed genes in (**A**) C57BL/6 vs. BALB/c cDC1 and cDC2 subsets, (**B**) cDC1 vs. cDC2 of BALB/c and C57BL/6 mice, (**C**) cDC1 isolated from ECTV-infected BALB/c and C57BL/6 mice at 5 dpi vs. control uninfected animals, and (**D**) cDC2 separated from ECTV-infected BALB/c and C57BL/6 mice at 5 dpi vs. control uninfected animals. The volcano plots show the threshold for statistical significance (unpaired Student’s *t*-test) of *p*-value = 0.05 (log10 of the *p*-value; horizontal line) and a fold change of −2 and +2 (log2; vertical dotted lines). The number of genes that are down (green)- and up (red)-regulated at least twofold and have a *p*-value less than 0.05 are at the upper-left and upper-right corners, respectively. Venn diagrams illustrating the overlap of down-regulated (**E**) and up-regulated (**F**) genes in cDC1 and cDC2 isolated from ECTV-infected BALB/c and C57BL/6 mice at 5 dpi. The numbers in red, grey, green, and blue modules represent the number of differentially expressed genes in BALB/c cDC1, C57BL/6 cDC1, BALB/c cDC2, and C57BL/6 cDC2, respectively.

Next, we sought to understand the molecular signatures driving the observed changes in cDC subsets by assessing the expression of selected genes involved in immune functions of cDC1 and cDC2 in spleens of susceptible and resistant strains of mice during early stages of mousepox. There were notable differences in maturation/activation gene expression among various cDC subpopulations between uninfected BALB/c and uninfected C57BL/6 mice (Figure 5A), as well as within each of these uninfected mouse strains between cDC1 and cDC2 subsets (Figure 5B). Furthermore, ECTV infection in BALB/c and C57BL/6 mice led to the down-regulation of 10 and 12 maturation genes, respectively, and the up-regulation of 5 and 4 genes, respectively, in the cDC1 subpopulation (Figure 5C). Within the cDC2 subpopulation, ECTV infection led to a decrease in the expression of 14 and 16 maturation genes, and increased the expression of 17 and 10 genes in BALB/c and C57BL/6 mice, respectively (Figure 5D). In the splenic cDC1 and cDC2 of both mouse strains, ECTV down-regulated Cd209a expression (Figure 5C) and up-regulated Ifng, Irf7, and Stat1 expression (Figure 5D). However, Ifng and Irf7 up-regulation in cDC subsets was higher in BALB/c mice compared to that in C57BL/6 mice. In the cDC2 subset of both mouse strains, ECTV infection resulted in the down-regulation of Cd74, Cxcl12, and Tgfb1, and the up-regulation of Ccl12, Cxcl10, Il12b, Il15, and Dhx58. The up-regulation of Ccl12, Cxcl10, Il12b, and Dhx58 was higher in BALB/c cDC2 compared to C57BL/6 cDC2, whereas the up-regulation of Il15 was comparable (2.42 vs. 2.22). The number of genes similarly down-regulated (Figure 5E) or up-regulated (Figure 5F) in cDC1 and cDC2 from both mouse strains at 5 dpi with ECTV are depicted in Venn diagrams.

**Figure 6:**
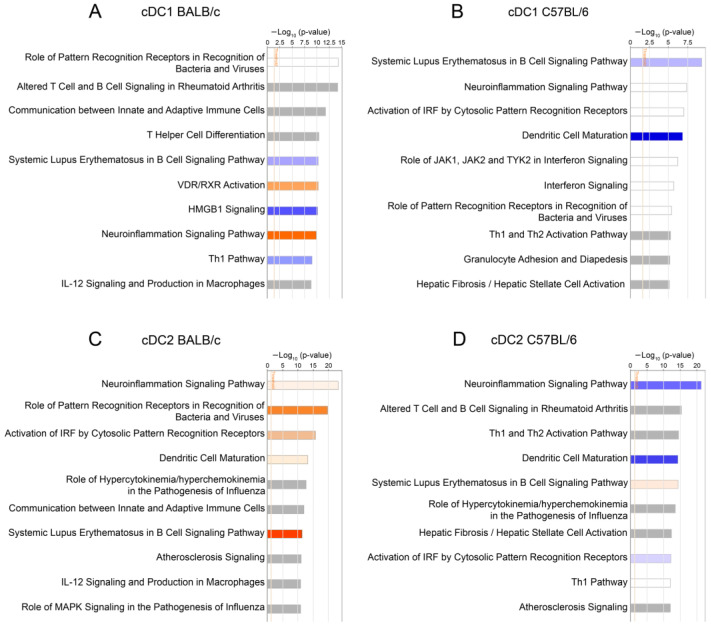
Top ten canonical pathways identified via ingenuity pathway analysis (IPA) of differentially expressed genes in BALB/c cDC1 (**A**), C57BL/6 cDC1 (**B**), BALB/c cDC2 (**C**), and C57BL/6 cDC2 (**D**) between ECTV-infected and control mice. Blue bars: negative z-score; orange bars: positive z-score; gray bars: no activity pattern available. The orange line shows the default *p*-value significance threshold of 0.05 (unpaired Student’s *t*-test).

Based on differentially expressed genes between cDC1 and cDC2 of respective mouse strains, IPA identified the top canonical pathways enriched in cDC subsets of both mouse strains during the early stages of mousepox (Figure 6). At 5 dpi in cDC1 of BALB/c mice, VDR/RXR Activation and Neuroinflammation Signaling Pathway were significantly activated, whereas HMGB1 Signaling and Th1 Pathway were predicted to be inhibited (Figure 6A). In cDC1 of C57BL/6 mice, Dendritic Cell Maturation was significantly inhibited (Figure 6B) similarly to cDC2 of C57BL/6 mice (Figure 6D). In contrast, in cDC2 of BALB/c mice, Dendritic Cell Maturation was predicted to be activated similarly to the Role of Pattern Recognition Receptors in Recognition of Bacteria and Viruses and Activation of IRF by Cytosolic Pattern Recognition Receptors (Figure 6C). These data suggest that cDC subsets from BALB/c mice show a higher level of activation and maturation compared to their counterparts from C57BL/6 mice.

### 3.6. cDC1 and cDC2 from C57BL/6 Mice Are More Potent in Stimulating the Th1 Immune Response Than Those from BALB/c Mice during Early Stages of Mousepox

**Figure 7:**
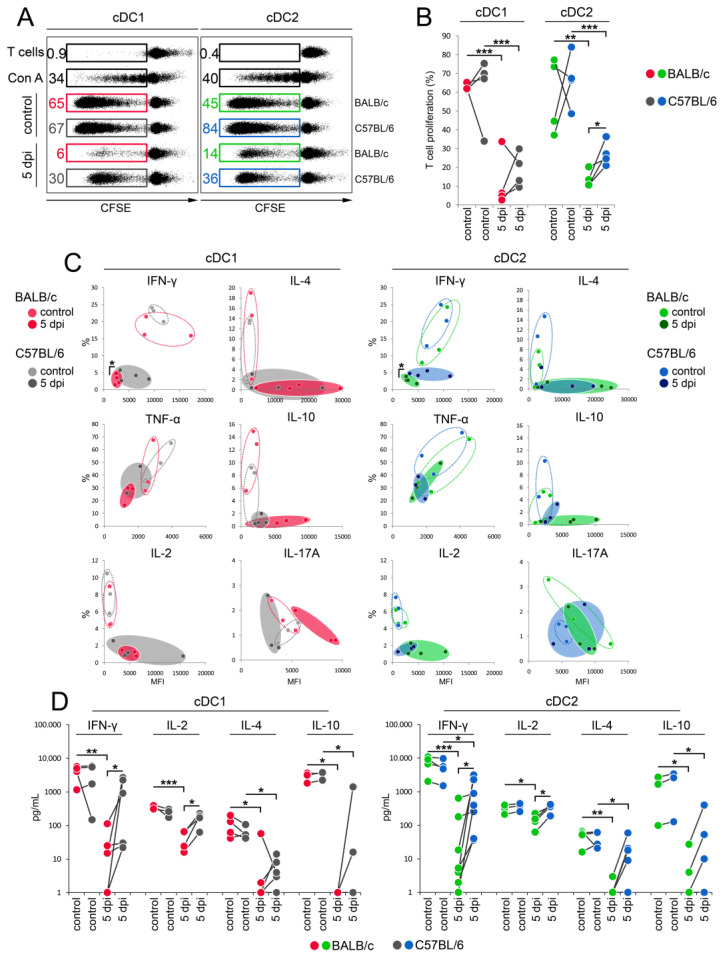
Diagram showing that ECTV infection decreases the ability of DC1 and cDC2 from BALB/c and C57BL/6 mice to stimulate proliferation and activation of allogeneic CD4^+^ T cells, but still both cDC subsets from resistant mice stimulated a greater Th1 cytokine immune response than did those of susceptible mice. The stimulatory ability of cDC subsets from control (uninfected) and ECTV-infected BALB/c and C57BL/6 mice at 5 dpi was detected from the allogeneic mixed lymphocyte reaction (MLR) in DC:T cell ratio = 1:5. Briefly, 2 × 10^5^ CFSE-labeled or unlabeled T cells from C3H mice were mixed with 4 × 10^4^ cDC subsets from ECTV-uninfected or -infected BALB/c and C57BL/6 mice and incubated for 5 days. (**A**) Representative flow cytometry dot plots showing the proliferation of CD4^+^ T cells in total CD4+ T cells, as measured via CFSE loss. Numbers show the percentage of CFSE^low^ proliferating T cells. Con A—concanavalin A. (**B**) Graph showing individual data of the percentage of CFSE^low^ CD4^+^ T cells from four independent experiments. Significant differences are indicated by horizontal bars between two dependent sets of data (unpaired Student’s *t*-test or Mann–Whitney U-test: * *p* < 0.05, ** *p* < 0.01, and *** *p* < 0.001). (**C**) The capacity of cDC1 and cDC2 subsets to stimulate the production of IFN-γ, TNF-α, IL-2, IL-4, IL-10, and IL-17A by allogeneic CD4^+^ T cells. Graphs show individual data (*n* = 3–4) plotted against the percentage of CD4^+^ T cells expressing a particular cytokine and mean fluorescent intensity (MFI) of this population, as determined via intracellular staining and flow cytometry analysis. Hatched and filled circles mark the distribution of individual data within a particular cDC subset from control and infected animals (5 dpi), respectively. Significant differences in MFI and the percentage of IFN-γ-producing cells are denoted by horizontal bars between two circles (unpaired Student’s *t*-test: * *p* < 0.05). (**D**) Diagrams representing the mean concentration values (pg/mL) of IFN-γ, IL-2, IL-4, and IL-10 produced by allogeneic CD4^+^ T cells in MLR with cDC1 or cDC2 from uninfected or infected BALB/c or C57BL/6 mice at 5 dpi. Significant differences are indicated by horizontal bars between two dependent sets of data (unpaired Student’s *t*-test or Mann–Whitney U-test: * *p* < 0.05, ** *p* < 0.01, and *** *p* < 0.001).

Finally, we addressed the ability of cDC1 and cDC2 subpopulations to stimulate T cell proliferation and the Th1 immune response in BALB/c and C57BL/6 mice during early stages of mousepox. Both cDC subpopulations from uninfected animals induced the strong proliferation of C3H T cells. The ECTV infection of BALB/c and C57BL/6 mice resulted in a statistically significantly (*p* < 0.05) reduced capacity of the cDC1 and cDC2 subpopulations to stimulate CD4^+^ T cells compared to control cells; however, the viral inhibitory effect was more pronounced in cDCs of BALB/c mice (Figure 7A,B). A very similar trend was observed for the ability of cDC subtypes to stimulate Th1 and Th2 cytokine production by allogeneic CD4^+^ T cells (Figure 7C,D). As determined by intracellular staining with flow cytometry and/or ELISA, under control conditions, both cDC subsets from both mouse strains induced the production of IFN-γ, TNF-α, IL-2, IL-4, IL-10, and IL-17A by allogeneic CD4^+^ T cells at a similar level. Meanwhile, ECTV infection resulted in a decrease in the ability of cDC1 and cDC2 to stimulate the production of cytokines by T cells in both mouse strains, while the inhibitory effect was more pronounced in cDC subsets of BALB/c mice. cDC1 and cDC2 from infected C57BL/6 mice stimulated a statistically significantly (*p* < 0.05) higher production of IFN-γ and IL-2 by C3HN T cells than did cDC1 and cDC2 from infected BALB/c mice (Figure 7C,D). These data indicate that in the early stages of mousepox, cDC1 and cDC2 subsets of both mouse strains show some impairments in their ability to elicit cytokine T cell responses; however, cDCs of resistant C57BL/6 mice are still more effective in stimulating Th1 responses than those from BALB/c mice.

## 4. Discussion

Different cDC subsets are specialized in preferentially polarizing Th immune responses: cDC1 in Th1 and cDC2 in Th2 [8]. However, this specialization can be influenced by factors such as the maturation stage, the activation of certain transcription factors, and the production of specific cytokines by cDCs [37]. Viruses can manipulate the immune response and have their persistence enhanced by altering the innate and adaptive immune functions of cDCs, thus influencing the pathogenic outcome of infection and the severity of the disease [38]. In this study, we compared the impact of ECTV infection on the Th-polarizing functions of cDC subsets derived from mice with different levels of genetic resistance to lethal mousepox (resistant C57BL/6 and sensitive BALB/c). This comparison aims to elucidate potential interstrain differences in the ability of cDCs to stimulate and direct the development of an adaptive immune response.

**Figure 8:**
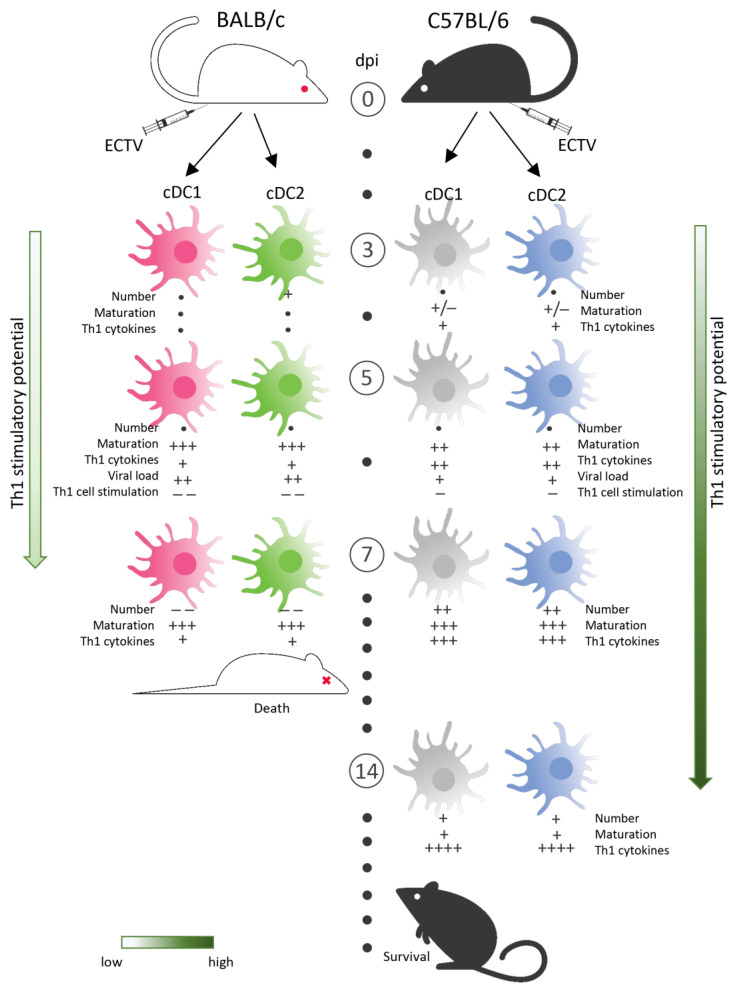
Schematic diagram of the kinetics of splenic cDC1 and cDC2 subset activation in BALB/c (susceptible) and C57BL/6 (resistant) mice during mousepox. ECTV infects both subsets of splenic cDCs in BALB/c and C57BL/6 mice, but cDC subsets from susceptible mice exhibit a higher viral load than that of those from resistant mice during early stages of mousepox. Susceptible mice show a reduced number of cDC1 and cDC2 in the spleen, which additionally produce lower levels of Th1-polarizing cytokines than those of resistant mice. However, cDC subsets from BALB/c mice show a higher maturation profile than that of those from C57BL/6 mice during the acute phase of mousepox. This phenomenon suggests that cDCs from susceptible BALB/c mice have a lower potential to stimulate a protective Th1-type response, resulting in the development of full-blown mousepox and death. cDC subsets from resistant mice have increased potential to stimulate a Th1-type response, resulting in survival and recovery from a primary ECTV infection. (+) means an increase compared to the control; (−) means a decrease compared to the control; (•) means no change compared to the control. The number of (+) and (−) indicates the intensification of a given process. dpi—days post infection.

Our results showed that following footpad ECTV challenge, the number of cDC1 was similar in spleens of both mouse strains during the first days of infection (from 3 to 5 dpi). cDC2 cells gradually began to decrease after 5 dpi in BALB/c mice, while in C57BL/6 mice, the number of cDC2 cells begun to increase gradually from 5 dpi and reached the maximum number at 7 dpi (Figure 8). In contrast, BALB/c mice showed a dramatic decrease in the number of both cDC subpopulations, probably due to severe organ necrosis at this time point of infection [39,40]. Moreover, virus re-isolation from purified splenic cDC subsets showed that at 5 dpi, cDC1 and cDC2 from BALB/c mice contained a higher viral load than did their C57BL/6 counterparts. In the scientific literature, there are data showing different susceptibilities of DCs from different mouse strains to infection with a particular infectious agent [26]. As an example, DCs from susceptible SJL mice, but not from resistant C57BL/6 mice, have been shown to be extremely permissive to Theiler murine encephalomyelitis virus (TMEV) infection [26]. In the context of mousepox, ECTV has been shown to reach vital organs such as the spleen and liver more quickly and in larger quantities in susceptible mouse strains compared to resistant C57BL/6 mice [17], as the latter strain harbors multiple nonspecific and specific immune mechanisms in the dLNs that limit the spread of the virus to the spleen [28,29,41,42]. The above observation could explain the difference in viral titers in cDC subsets between BALB/c and C57BL/6 mice.

In this study, we demonstrate that despite productive infection, both cDC subpopulations were able to mature in both mouse strains with mousepox. Unexpectedly, the maturation degree at 5 dpi was even higher in more infected cDC1 and cDC2 subpopulations from susceptible BALB/c mice, compared to that in their less infected counterparts among resistant C57BL/6 mice (Figure 8). This observation is in contradiction to that from our previous in vitro studies with GM-BM cells, in which ECTV infection led to the severe functional impairment of these cells [32,34]. The cell type, but also to the virus load, may be responsible for such inconsistency between in vitro and in vivo studies [43]. Additionally, an in vivo study has shown the impaired maturation of CD11c^+^ cells in the dLNs and spleens of BALB/c mice at 5 dpi following footpad ECTV infection; however, in this study, individual cDC subpopulations were not distinguished [44]. The in vivo maturation of splenic DCs was also observed in the context of other orthopoxvirus infections, including vaccinia virus (VACV) [43]. However, at 2 dpi, the level of MHC II on the surface of DCs was decreased, although the expression of MHC I and co-stimulatory molecules remained elevated [43]. In our study, at 7 days post-infection (dpi), the cDC2 subpopulation in BALB/c mice showed the reduced expression of MHC II molecules. This suggests the compromised ability of cDC2 to present antigens via MHC II molecules in susceptible mice during the acute phase of mousepox.

It is noteworthy that despite the lower maturation profile compared to that of BALB/c mice, both cDC subsets from resistant C57BL/6 mice produced higher levels of Th1-polarizing cytokines during the early stages of mousepox (3–5 dpi). The production of Th1-polarizing cytokines peaked mainly at 5 dpi in the case of cDC2 and at 7 dpi in the case of cDC1 (Figure 8). In contrast to the cDC1 subpopulation in BALB/c mice, that of cDC1 in C57BL/6 mice was the main producer of IFN-γ and CCL3 at 7 dpi. It is not excluded that cDC1 and cDC2, especially in resistant C57BL/6 mice, are substantial sources of IFN-γ and CCL3 not only during the early stages of mousepox, but also during recovery, since at 14 dpi most cDC1 cells produced this cytokine. This observation aligns with prior research showing that both murine and human cDC1 and cDC2 cells are key producers of IFN-γ in response to viruses [26,45,46] and various other intracellular pathogens or their components [47,48,49,50]. It is possible that the heightened production of Th1-polarizing cytokines by cDC subpopulations, particularly cDC1, in C57BL/6 mice during the early stages of mousepox could positively influence the timing, kinetics, and intensity of the protective Th1 immune response in resistant strains. cDC1 cells might surpass cDC2 in Th1 polarization efficiency as they produce both IFN-γ and IL-12. These cytokines synergistically induce the complete polarization of CD4 T cells into Th1 cells [51] and collaborate with other immune cells to stimulate the Th1 response [52,53,54,55,56].

The results of our study also indicated that, during early stages of mousepox (until 5 dpi), there is a difference in activation/maturation kinetics between cDC1 and cDC2 subsets, from both strains of mice. cDC2 subsets are strongly stimulated and mature earlier than do cDC1 subsets. Within the spleen, as well as dLNs, there is a spatiotemporal cellular organization of cDC subsets under steady-state and infection conditions, which may have implications for the induction of immunity [8,57,58]. In the spleen, steady-state resident cDC1 cells are mainly found within the central T cell zone of the white pulp (or PALS), and the antigen must be transferred to them from migratory cDCs or conduits [59]. Migratory cDC1 cells appear within the red pulp and marginal zone and are able to migrate to the T cell areas of the white pulp in response to microbial stimulation. In homeostasis, cDC2 cells, which are the major cDC subpopulation in the spleen, reside within the red pulp and marginal zone, closer to the B cell follicles; however, they are most enriched in the marginal zone bridging channels (the interfaces of the white pulp and the red pulp), where they sample large, particulate circulating antigens. In response to inflammation, cDC2 rapidly relocate to the splenic T cell zone for the stimulation of CD4^+^ T cells and antibody production [8,57,58]. The spleen has open blood circulation, and blood is released into the marginal zone through terminal arterioles, or directly into the red pulp [60]. Because cDC2 cells are located in a blood-exposed bridging channel zone, these cells effectively capture antigens from the blood for subsequent presentation to T cells [61]. Therefore, cDC2 cells may acquire blood-borne antigens and pathogens more easily and faster than resident cDC1 cells do, and it might influence the timing of antigen sampling according to different cDC subsets. Such anatomical regulation of antigen uptake by different cDC subsets is observed within dLNs [8], in which general features of cDC subset localization and migration are shared with the spleen [58]. The influence of the spatiotemporal organization of cDC subsets on blood-borne antigen sampling in the context of ECTV infection requires further exploration.

Our last question concerned the ability of cDC1 and cDC2 subsets isolated from ECTV-infected BALB/c and C57BL/6 mice to directly prime and polarize CD4^+^ T cells towards Th1/Th2. Despite the maturation of both DC subsets in BALB/c and C57BL/6 mice during ECTV infection, these cells were highly limited in their capacity to activate proliferation and cytokine production, mainly that of Th1, by a large number of allogeneic CD4^+^ T cells compared to control cells in both strains (Figure 8). Therefore, the proliferation and cytokine response of CD4^+^ T cells stimulated by cDC1 and cDC2 cells isolated from infected animals of both mouse strains deviated from that stimulated by intact control cDC subsets. Nevertheless, cDC1 and cDC2 from resistant mice were less affected by the virus, and, therefore, were able to stimulate both proliferation and Th1 cytokine responses more efficiently than their BALB/c counterparts were. It is not known whether or not cDC populations from different mouse strains had different susceptibilities to ECTV infection, but our previous in vitro studies have demonstrated that GM-BM cells are susceptible to ECTV infection to a comparable extent, regardless of the mouse strain’s susceptibility/resistance to mousepox [32]. It is also unlikely that more infected cDCs from susceptible BALB/c mice would release higher amounts of infectious virus into the culture medium during DC–T cell co-culture, therefore affecting allogeneic T cell stimulation more drastically, since mitomycin C completely inhibits poxvirus replication in exposed cells [62,63]. DCs from infected resistant C57BL/6 mice are more potent at stimulating allogeneic Th1 cells than are BALB/c DCs; however, it is not clear whether or not such stimulation would be sufficient in vivo to generate an optimal Th1 response alone or in cooperation with other immune cells.

## 5. Conclusions

Our data reveal, for the first time, that both cDC1 and cDC2 are activated and mature following in vivo ECTV infection in BALB/c and C57BL/6 mice. However, cDC subpopulations in susceptible mice achieve a higher level of maturation compared to their counterparts in resistant mice. During the acute phase of mousepox, cDC1 and cDC2 subpopulations lose their full ability to polarize responses involving Th1 lymphocytes, and this phenomenon is more pronounced in the susceptible mouse strain, rendering it unable to develop protective Th1 immunity. Collectively, our results show that in the early stages of ECTV infection, especially in susceptible BALB/c mice, cDC subpopulations, despite their maturation, are unable to perform their antigen-presenting functions; therefore, the host experiences transient immunosuppression due to the inhibition of the development of the protective antiviral Th1 immune response. This also suggests that an alteration in adaptive immune functions of cDCs may be a common tactic used by various viruses to favor their own persistence, dissemination, and survival in the infected host. Therefore, a full and complete understanding of poxviral interaction with cDCs will help to design effective vaccination strategies for preventing poxviral infections.

## Figures and Tables

**Table 1:** Characterization of splenic mouse cDC1 and cDC2 subpopulations.

Feature	cDC1	cDC2
location	marginal zone	T-cell areas of PALS
phenotype	CD8α^+^CD11b^−^	CD8α^−^CD11b^+^
	XCR1^+^	SIRPα^+^
	DNGR-1^+^	CD4^+/−^
	CD205^+^	
	CD207^+^	
Th cell differentiation	Th1	Th2
human homologues	CD141^+^ DCs	CD1^+^ DCs

cDC—conventional dendritic cell; DNGR-1—dendritic cell NK lectin group receptor 1; SIRPα—signal regulatory protein α; Th—T helper; PALS—periarterial lymphoid sheath; XCR1—X-C motif chemokine receptor 1

**Table 2:** The examples of DC engagement in the polarization of the Th1 or Th2 immune responses depending on host resistance/susceptibility to the infection.

Pathogen	Type of DCs	Resistance	Susceptibility	Ref.
*Leishmania major*	CD11b^+^ DCs ex vivo	Th1 response in B10.D2 mice	Th2 response in BALB/c mice	[23]
	BMDCs in vitro	Th1 response in B10.D2 mice	Th2 response in BALB/c mice	[23]
*Listeria monocytogenes*	cDC in vivo	Higher expression of IL-12p40 in cDCs of C57BL/6 mice	Higher expression of CCL2 in cDCs of BALB/c mice	[24]
*Rickettsia conorii*	BMDCs	High ability to stimulate IFN-γ-producing Th1 cells from C57BL/6 mice	Low ability to stimulate IFN-γ-producing Th1 cells from C3H mice	[25]
Theiler’s murine encephalomyelitis virus (TMEV)	DCs in vitro and in vivo	Lack of conversion from Th1 into Th2 in C57BL/6 mice	Conversion from Th1 into Th2 in SJL mice	[26]

## Data Availability

Research data are available upon request from L.S.-D.
